# P2X_7_R modulation of visually evoked synaptic responses in the retina

**DOI:** 10.1007/s11302-016-9522-7

**Published:** 2016-07-08

**Authors:** Seetal Chavda, Philip J. Luthert, Thomas E. Salt

**Affiliations:** 1 Visual Neuroscience, UCL Institute of Ophthalmology, London, EC1V 9EL UK; 2 Ocular Biology and Therapeutics, UCL Institute of Ophthalmology, London, EC1V 9EL UK; 3 NIHR Biomedical Research Centre in Ophthalmology, London, EC1V 9EL UK

**Keywords:** P2X_7_ receptor, Adenosine 5′-triphosphate, Retina, Neuromodulation

## Abstract

P2X_7_Rs are distributed throughout all layers of the retina, and thus, their localisation on various cell types puts into question their specific site(s) of action. Using a dark-adapted, ex vivo mouse retinal whole mount preparation, the present study aimed to characterise the effect of P2X_7_R activation on light-evoked, excitatory RGC ON-field excitatory post-synaptic potentials (fEPSPs) and on outer retinal electroretinogram (ERG) responses under comparable conditions. The pharmacologically isolated NMDA receptor-mediated RGC ON-fEPSP was reduced in the presence of BzATP, an effect which was significantly attenuated by A438079 and other selective P2X_7_R antagonists A804598 or AF27139. In physiological Krebs medium, BzATP induced a significant potentiation of the ERG a-wave, with a concomitant reduction in the b-wave and the power of the oscillatory potentials. Conversely, in the pharmacologically modified Mg^2+^-free perfusate, BzATP reduced both the a-wave and b-wave. The effects of BzATP on the ERG components were suppressed by A438079. A role for P2X_7_R function in visual processing in both the inner and outer retina under physiological conditions remains controversial. The ON-fEPSP was significantly reduced in the presence of A804598 but not by A438079 or AF27139. Furthermore, A438079 did not have any effect on the ERG components in physiological Krebs but potentiated and reduced the a-wave and b-wave, respectively, when applied to the pharmacologically modified medium. Therefore, activation of P2X_7_Rs affects the function in the retinal ON pathway. The presence of a high concentration of extracellular ATP would most likely contribute to the modulation of visual transmission in the retina in the pathophysiological microenvironment.

## Introduction

The P2X_7_ receptor (P2X_7_R) is the most diverse receptor subtype of the P2X family in both structure and function [1]. ATP-gated P2X_7_Rs have been detected in neurons and glia, in both inner and outer synapse-rich and nuclear layers of the retina. They are functionally expressed by retinal ganglion cells [2–10] and Müller glial cells [11], and their presence on amacrine, horizontal [7, 12, 13] and microglial cells [4, 7] has also been suggested. The upregulation of P2X_7_R expression and activation by extracellular ATP is associated with the elevation of intracellular calcium and subsequent photoreceptor and retinal ganglion cell (RGC) death, thus implicating a role for P2X_7_Rs in retinal degenerations such as retinitis pigmentosa, glaucoma, age-related macular degeneration (AMD) and retinopathies [14, 15].

The P2X_7_R is relatively insensitive to its extracellular agonist, adenosine 5′-triphosphate (ATP), and exhibits a biphasic agonist-evoked response, dependent upon the agonist exposure time [16]. Similar to other P2X subtypes, a brief agonist application induces a reversible, transient inward current through a non-selective ion channel, permeable to small cations. Repeated or prolonged agonist application gives rise to a sustained current, which could ultimately lead to cell death, assumed to be associated with the opening of a large pore that is permeable to molecules of high molecular weight (<900 Da) [16, 17].

Although P2X_7_R function in retinal pathophysiology is becoming established, the role of these receptors in synaptic function in the healthy retina has remained largely unexplored, until now. The specific localisation of P2X_7_Rs on neurons of the vertical rod-mediated pathway suggests that these receptors may have an important role in modulating scotopic visual responses under physiological conditions [7, 13]. Under physiological conditions, in vivo electroretinogram (ERG) studies have shown alterations to both outer and inner retinal function, in response to short-term administration of the P2XR agonist, BzATP, in the rat retina [7, 12, 13]. However, the specific contribution of P2X_7_Rs to the BzATP-mediated modulation of retinal function remains elusive. A further complexity is that P2X_7_Rs are distributed throughout all layers of the retina, adding to the uncertainty of the specific site(s) of action for these receptors in modulating neurotransmission within the retina. In line with this notion, it is unclear from the sole use of ERG recordings whether P2X_7_Rs also directly modulate retinal ganglion cell synaptic function.

A large body of evidence suggests a role for neuronal P2X_7_Rs in the modulation of neurotransmitter release and subsequent regulation of synaptic function [17]. P2X_7_R-mediated neurotransmitter release has been detected in the cerebellum [18], cerebral cortex and midbrain preparations [19–21], hippocampus [22] and neuromuscular junction [23]. Indeed, similar mechanisms may be at play within the retina, and the expression of P2X_7_Rs on Müller glia and microglia also implicates a contribution to neuromodulation by non-neuronal sources to visual processing, by the activation of these receptors.

The aim of the present study was to investigate the effect of P2X_7_R activation on visually evoked synaptic responses at the level of the photoreceptor, the ON bipolar cell, and RGC. We have performed this using a dark-adapted ex vivo mouse retinal whole mount preparation to record extracellular, light-evoked retinal responses and with the use of selective P2X_7_R compounds. Our findings suggest that functional P2X_7_Rs are present throughout the visual pathway in the retina and that their activation leads to modulation of visual responses.

## Materials and methods

### Animals

Adult (4–12 weeks) C57BL/6 mice (Harlan Laboratories, UK) were housed on a 12-h light/dark cycle, in the Biological Resources Unit, Institute of Ophthalmology, University College London. The animals were given unlimited access to food and water. All procedures were in accordance with the UK Animals (Scientific Procedures) Act 1986 and approved by the UK Home Office and the University College London local ethics committee.

### Preparation of the ex vivo mouse retinal whole mount

All procedures were carried out in low-light conditions and red light was used for illumination, unless otherwise stated. Animals were killed by cervical dislocation and decapitation. The eyes were rapidly removed and placed into an ice-cold, oxygenated sucrose Krebs medium containing the following (mM): sucrose 202, KCl 2, KH_2_PO_4_ 1.25, MgSO_4_ 10, CaCl_2_ 0.5, NaHCO_3_ 26 and glucose 10. The eyes were placed in a Petri dish containing sucrose Krebs medium, under a dissection microscope, and the optic nerve and extra-ocular muscle tissue were removed from the sclera. A cut was made at the ora serrata, and the cornea and iris were removed, followed by the removal of the lens and vitreous. The open eye-cup, consisting of the intact retina, retinal pigment epithelium and sclera, was cut into quadrants to allow it to lie flat, as a whole mount preparation, with the integrity of the retinal circuitry maintained and attached to the retinal pigment epithelium.

### Perfusion and recording chamber

The retinal whole mount, ganglion cell layer side up, was transferred to a blacked-out interface recording chamber. The retina was held partially submerged and superfused with oxygenated Krebs medium containing the following (mM): NaCl 124, KCl 2, KH_2_PO_4_ 1.25, MgSO_4_ 1, CaCl_2_ 2, NaHCO_3_ 26 and glucose 10, at 0.4 ml/min and 36 ± 0.2 °C. The whole mount was left to recover for approximately 60 min prior to experimentation.

### Response acquisition and recording electrode

Transretinal electroretinogram (ERG) and RGC field excitatory post-synaptic potential (fEPSP) recordings were made via Krebs medium-filled extracellular recording electrodes (2–8 MΩ), pulled from borosilicate capillaries (1.2 × 0.69 mm, Clark Electromedical Instruments). Responses were recorded with an Axoprobe-1A amplifier (Axon Instruments), digitised (5 kHz) via a CED1401 interface and stored on a computer with Spike2 software (Cambridge Electronic Design Ltd., Cambridge, UK). Mains-related noise (50 Hz) was eliminated with a Humbug noise reduction system (Quest Scientific).

**Fig. 1:**
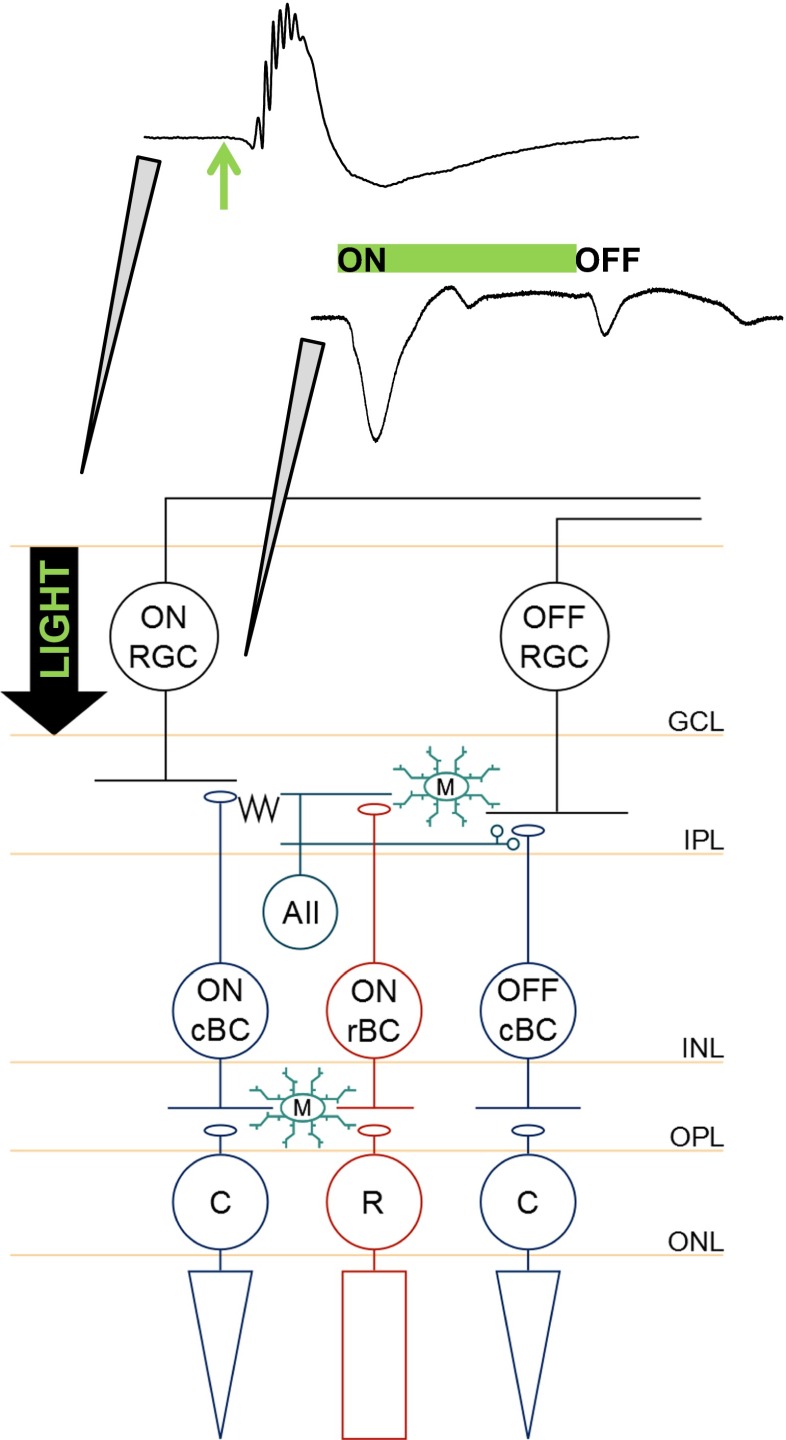
Placement of recording electrodes for acquisition of transretinal electroretinogram and retinal ganglion cell fEPSP responses. Schematic illustrates retinal circuitry and the respective positions of the recording electrode for the electrophysiological experiments. The retinal whole mount was placed in the bath, ganglion cell layer side up. The full-field light flash stimulus was placed above the slice, and the *arrow* shows the direction in which the light travelled. *Top trace*, Typical transretinal electroretinogram response recorded by lowering the electrode onto the surface of the retinal whole mount, without breaking into the layers. The response was repeatedly elicited by a single 10-ms duration flash (*green arrow*), with a 3-s interval. *Bottom trace*, Retinal ganglion cell fEPSPs were recorded by placing the electrode in the ganglion cell layer. The *green bar* represents the duration of the stimulus (1 s), which was repeated at 3-s intervals. Upon stimulus onset, an ON retinal ganglion cell fEPSP was generated. Stimulus offset produced an OFF retinal ganglion cell fEPSP. *ONL* outer nuclear layer, *OPL* outer plexiform layer, *INL* inner nuclear layer, *IPL* inner plexiform layer, *GCL* ganglion cell layer, *M* microglia, *cBC* cone bipolar cell, *rBC* rod bipolar cell, *AII* AII amacrine cell

To obtain transretinal ERG responses, the recording electrode was manually lowered onto the surface of the retinal whole mount, without breaking into the retinal layers. For RGC fEPSP acquisition, a MS314 electronic micromanipulator (Marzhauser-Wetzler) was used to further lower the electrode vertically, from the surface of the whole mount in 5–10 μm steps, at a rate of 1 μm/step, every 10–15 s until breakthrough to the ganglion cell layer, where recordings were obtained. Figure 1 illustrates the positions of the recording electrodes for acquisition of ERG and fEPSP responses.

### Visual stimulation and characterisation of responses

The retinal whole mount was stimulated with a full-field, green light-emitting diode (LED; *λ* = 562 nm) via a fibre-optic light guide set 2 cm above the preparation. ERG responses were stimulated with a 10-ms duration flash, repeated every 3 s. RGC fEPSPs were stimulated with a 1-s duration flash, repeated every 3 s. For all experiments, the light stimulus was set to an intensity at which sub-maximal responses were elicited, typically within the range of −1.47 to −0.96 log cd s/m^2^.

Upon stimulation, the transretinal ERG exhibited a characteristic waveform, composed of the negative a-wave, a prominent and large-amplitude positive b-wave and oscillatory potentials superimposed on the leading edge of the b-wave, qualitatively similar to rodent rod ERGs obtained in vivo [24]. For the RGC fEPSP experiments, the onset and termination of the light stimulus produced negative RGC ON- and OFF-fEPSPs, respectively, similar to those described previously [25, 26].

### Pharmacological investigations

Pharmacological compounds were added into the bathing medium, which continuously superfused the retinal preparation through the course of each experiment. All ERG experiments were carried out in either physiological, low Mg^2+^, or a pharmacologically modified Krebs medium (hereafter referred to as PMSTN) that was composed of the following (mM): NaCl 124, KCl 2, KH_2_PO_4_ 1.25, CaCl_2_ 2, NaHCO_3_ 26, glucose 10, NBQX 0.01, picrotoxin 0.1, strychnine 0.005 and tetrodotoxin 0.0001. AMPA, GABA_A/C_ and glycine receptors and sodium channels were blocked with NBQX, picrotoxin, strychnine and tetrodotoxin, respectively. The effect of P2X_7_R activation on synaptic transmission at rod photoreceptor-ON bipolar cell synapses was investigated under three different conditions: (1) physiological Krebs medium, (2) low Mg^2+^ Krebs medium and (3) PMSTN (as with the RGC experiments). Firstly, normal Krebs medium was used to explore the effect of P2X_7_R activation on the ERG under physiological conditions. PMSTN medium was used as it allowed comparison of the effect of P2X_7_R activation on the ERG and the ON-RGC fEPSP under similar conditions. Additionally, it also enabled us to identify whether an upstream mechanism in the outer retina could be influencing the effect further downstream at the level of the retinal ganglion cells. The use of the low Mg^2+^ perfusate enabled further characterisation of the role of P2X_7_Rs in the retina, as their activation is considered to be enhanced in the absence of divalent cations. All RGC fEPSP experiments were undertaken using the PMSTN medium, in order prevent action potential firing and to isolate and reveal the NMDA-mediated component of the RGC ON-fEPSPs.

Differences in protocols for investigating P2X_7_R function in ERG and RGC fEPSP experiments are highlighted where necessary. The P2X_7_R agonist, BzATP, was applied for 10 min. For the concentration-response investigation of BzATP on RGC ON-fEPSPs, no more than two applications of BzATP were used on one preparation, regardless of concentration. The same protocol was followed for the concentration-response investigation of adenosine on RGC fEPSP responses.

To test the effect of BzATP in the presence of the P2X_7_R antagonist A438079 on both ERG and RGC fEPSP responses, BzATP (300 μM) was added to the PMSTN medium for 10 min, followed by a 30–40-min washout period. A438079 was then added to the medium and allowed to superfuse the retina until a stable response was reached (∼20–30 min). BzATP was then co-applied with the antagonist-containing medium. In separate experiments, the effect of the P2X_7_R antagonists A804598 and AF27139 (both prepared in DMSO) on the BzATP-mediated effect on RGC fEPSPs was tested using the same protocol as for A438079. As a control, DMSO (0.1 %) was added to the PMSTN medium in these experiments.

### Digital filtering and analysis of ERG components

**Fig. 2:**
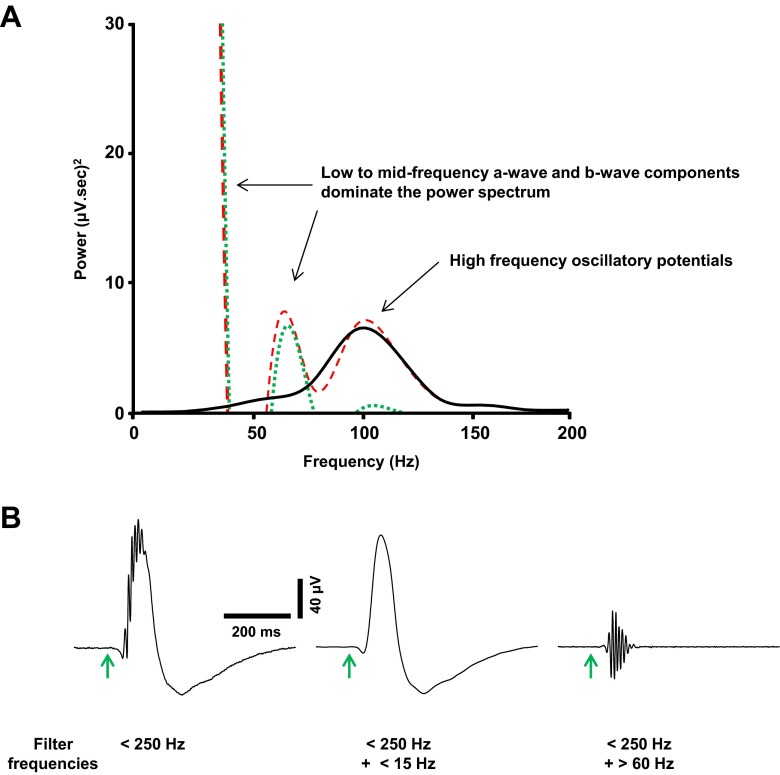
Digital filtering of transretinal electroretinogram responses. **a** Dark-adapted electroretinogram (ERG) power spectra (300 s averages) under various filter settings. The ERG was low-pass-filtered (<250 Hz; *dashed traces*), with the prominent a-wave and b-wave components within the low to mid frequency range. Further low-pass filtering (<15 Hz; *dotted traces*) eliminated the high-frequency component (oscillatory potentials). A high-pass filter (>60 Hz; *solid trace*) was applied after the first filter in order to isolate the high-frequency oscillatory potentials, which exhibited a peak frequency of approximately 100 Hz. **b** Waveform averages illustrate the effect of the filter settings on the shape of the ERG. Application of two orders of low-pass filter did not distort the waveform keeping the a-wave and b-wave intact. Application of the high-pass filter eliminated the a-wave and b-wave isolating the oscillatory potentials for further analysis. *Arrows* represent onset of flash stimulus (10 ms)

The ERG traces were analysed offline using Spike2 software (Cambridge Electronic Design, UK). Waveforms were initially low-pass-filtered (<250 Hz) to reduce contamination of the signal by high-frequency noise. A secondary low-pass filter (<15 Hz) was applied to isolate a smoothed ERG and eliminate the oscillatory potentials. Figure 2 shows no overall distortion in the power spectra or waveforms of the low-frequency ERG components (a-wave and b-wave), following two orders of low-pass filtering. The smoothed waveform ensured accurate measurement of the a-wave slope and b-wave amplitude without contamination from the oscillatory potentials.

The slope of the a-wave, which reflects photoreceptor activity [27], was measured between the onset and peak of the a-wave. The b-wave peak amplitude was measured from the peak of the a-wave to half the recovery phase of the b-wave and is known to reflect ON bipolar cell function [28]. Sweeps were analysed by averaging these parameters over 60 s, throughout the time course of the experiment. Drug effects on both response components were measured by calculating the mean ± SEM percentage change in the measured parameters (slope or amplitude), relative to control values. The implicit time of the b-wave, in particular, can provide a measure of photoreceptor sensitivity, whereas possible contamination of the a-wave by the onset of the b-wave must also be considered during analysis of the kinetics of these ERG components [29]. The latencies were calculated from stimulus onset to the peak amplitudes of the a-wave or b-wave, and values are shown as mean ± SEM implicit time.

Oscillatory potentials were analysed in the frequency domain. These high-frequency wavelets are believed to reflect inner retinal network function, particularly the activity of amacrine and ganglion cells [30]. A secondary high-pass filter (>60 Hz) was applied to isolate the oscillatory potentials from the first filtered waveform, as shown in Fig. 2. The oscillatory potentials exhibited a peak frequency of ∼90–110 Hz, which is comparable to that of dark-adapted mouse ERGs in vivo [31]. A fast Fourier transform was used to derive the power spectrum of the oscillatory potentials, from stimulus onset to the half-width of the b-wave recovery phase (∼240 ms). To analyse drug effects, average spectra were generated 120 s before, during and after drug application, and the mean ± SEM percentage change in peak power was compared with control values.

### Analysis of RGC ON-fEPSPs

RGC ON-fEPSP traces were also analysed offline using Spike2 software (Cambridge Electronic Design, UK). Responses were visualised as 3-s ‘sweeps’ and were analysed by measuring the average amplitude of the ON-fEPSPs over 60 s, throughout the time course of the experiment. The effect of pharmacological agents on the ON responses was measured by calculating the mean ± SEM percentage change in fEPSP amplitude, compared with control values.

### Statistical analysis

Data were imported from Spike2 to Excel (Microsoft, USA) for analysis and generating graphs. Statistical testing was carried out using GraphPad Prism (v.6 for Windows, CA, USA). For comparisons between baseline and drug effect on the responses, statistical significance was established using the Wilcoxon matched pairs test. Comparisons between treatment groups were carried out using the Mann-Whitney test. For all tests, statistical significance was observed if *P* < 0.05.

## Results

### Suppression of the retinal ganglion cell ON-fEPSP by BzATP and reduction of the effect by the selective and competitive P2X_7_ receptor antagonists A438079, A804598 and AF27139

**Fig. 3:**
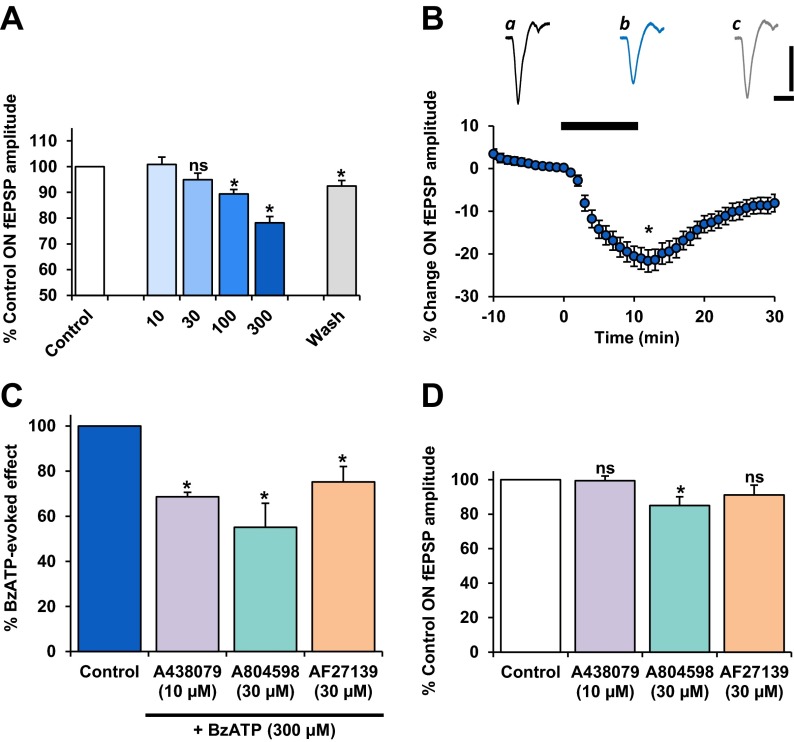
The effect of BzATP on the light-evoked ON-fEPSP. Responses are pharmacologically isolated, NMDAR-mediated. BzATP was applied (10 min) at concentrations of 10 (*n* = 2), 30 (*n* = 6), 100 (*n* = 6) and 300 μM (*n* = 21). Values are mean ± SEM percentage of pre-treatment control fEPSP peak amplitude. **a** BzATP elicited a concentration-related reduction of the ON-fEPSP. Washout of BzATP (300 μM) induced recovery of the ON-fEPSP. **b** Time course plot shows the effect of BzATP (300 μM) on the ON-fEPSP. Plotted values are mean ± SEM percentage change in fEPSP peak amplitude, relative to pre-treatment control. Representative traces (120 s averages) illustrate the effect of BzATP (300 μM) on the ON-fEPSP. *a* Control, *b* BzATP (300 μM), *c* wash. *Scale bars* = 400 ms, 20 μV. **c** Actions of selective P2X_7_R antagonists on the BzATP-induced suppression of the ON-fEPSP. The selective P2X_7_R antagonists, A438079 (*n* = 6), A804598 (*n* = 7) or AF27139 (*n* = 6), significantly attenuated the BzATP (300 μM)-mediated reduction of the ON-fEPSP. All three compounds exhibited similar potency in blocking P2X_7_R function at the concentrations tested. Note that in the presence of all antagonists, a large residual effect on the ON-fEPSP persisted with BzATP application. **d** Direct actions of selective P2X_7_R antagonists on the NMDAR-mediated ON-fEPSP. The ON-fEPSP was significantly reduced in the presence of A804598 (*n* = 7) but not by A438079 (*n* = 6) or AF27139 (*n* = 6) at the concentrations tested. *ns* not significant; *P < 0.05, compared to control

The effect of P2X_7_R activation on synaptic responses of RGC was investigated. All experiments were carried out in PMSTN medium. As shown in Fig. 3, BzATP was applied to the perfusate for 10 min, and the agonist-induced modulation of the ON-fEPSP was compared across concentrations ranging from 10 to 300 μM. There was no significant effect on the ON-fEPSP with low micromolar concentrations of BzATP. However, a concentration-related reduction in the ON-fEPSP peak amplitude was measured at higher micromolar concentrations. BzATP at 300 μM, the highest concentration tested, elicited a significant reduction in the ON-fEPSP to 78.1 ± 3 % of control (*P* < 0.05, *n* = 21) (Fig. 3a, b). BzATP elicited its effect within approximately 2–3 min of reaching the retina and was sustained throughout the duration of application (Fig. 3b(ii)). This effect was partially recoverable on washout to 92.4 ± 2 % of control (*P* < 0.05).

The effect of BzATP on the ON-fEPSP was assessed in the presence of the selective, competitive P2X_7_R antagonists A438079, A804598 or AF27139. Compared to the more classically used P2X_7_R antagonists, A438079 and A804598 are generally considered amongst the most potent suppressors of mouse P2X_7_R function, of those commercially available [32, 33]. AF27139 (Lundbeck, DK) is not yet commercially available, and A804598 has not been widely tested, but both have shown to potently block P2X_7_R-mediated cytokine release in cell culture preparations across species [33]. The less novel A438079 was chosen for this study as it has demonstrated potent suppression of P2X_7_R function in models of chronic pain, microglial neuroinflammation and neurodegenerative disorders in the spinal cord and brain [34–38]. The use of A438079 in ex vivo retinal whole mount preparations has not previously been reported, although the compound has proven potent in suppressing swelling-induced damage to isolated retinal ganglion cells [9].

Experiments were separate for each antagonist and were carried out in PMSTN medium. The effect of each antagonist on the BzATP-induced changes in the ON-fEPSP was directly compared to the preceding application of BzATP in the absence of the antagonist. A438079 (10 μM), A804598 (30 μM) and AF27139 (30 μM) all partially attenuated the BzATP-mediated reduction of the ON-fEPSP to 68.7 ± 2 %, 55.1 ± 11 % and 75.2 ± 7 % of control, when compared to the maximum effect of BzATP (*P* < 0.05 for all) (Fig. 3c).

To explore whether the release of endogenous ATP acting on P2X_7_Rs during visual stimulation affects ON-centre retinal ganglion cell activity, the effects of A438079, A804598 and AF27139, when applied alone, were investigated (Fig. 3d). The ON-fEPSP was reduced in the presence of A804598 to 85.0 ± 15 % of control (*P* < 0.05, *n* = 7) but was not significantly affected by A438079 or AF27139 at the concentrations tested.

### Adenosine potentiates the retinal ganglion cell ON-fEPSP

Extracellular ATP is rapidly degraded into its constituent adenosine by ectonucleotidases. It has previously been suggested that the actions of BzATP in the hippocampus are due to the effect of its breakdown product Bz-adenosine through the subsequent activation of A1 receptors [39]. Adenosine receptors are known to be expressed in the retina. Therefore, it was investigated whether the effect of BzATP on the RGC fEPSPs was mediated by the activation of adenosine receptors.

**Fig. 4:**
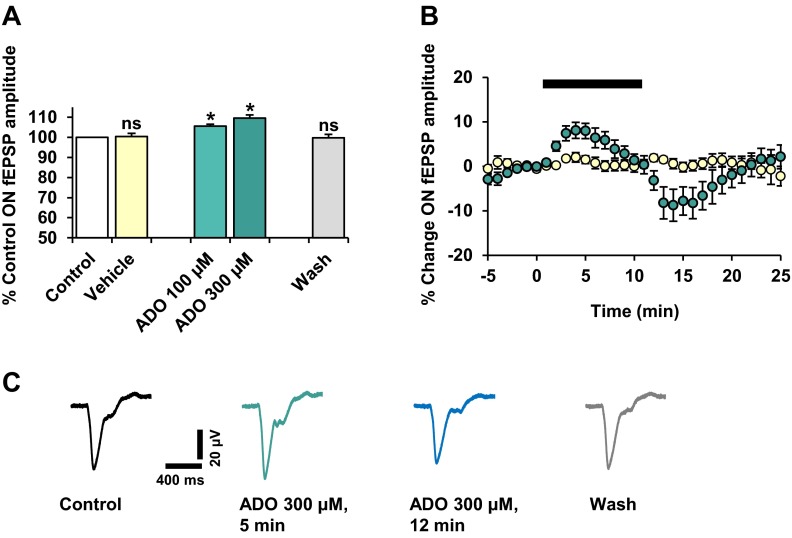
The effect of adenosine on the NMDAR-mediated RGC ON-fEPSP. Adenosine (ADO) was applied (10 min) at concentrations of 100 and 300 μM. *n* = 8 for both concentrations tested. **a** Values are mean ± SEM percentage of pre-treatment control fEPSP peak amplitude, taken ∼5–7 min after the start of adenosine application. The ON-fEPSP was reversibly and significantly potentiated by adenosine, in a concentration-related manner. Vehicle application elicited no effect on the ON-fEPSP. *ns* not significant; *P < 0.05, compared to control. **b** Time course plot shows the effect of adenosine (300 μM; *green*) and vehicle (*yellow*) on the ON-fEPSP. Plotted values are mean ± SEM percentage change in fEPSP peak amplitude relative to pre-treatment control. **c** Representative traces (120 s averages) illustrate the effect of adenosine (300 μM) on the ON-fEPSPs

The ON-fEPSP was significantly potentiated by adenosine, exhibiting a dose-related effect. For the highest concentration tested (300 μM), maximum potentiation was achieved within 3–5 min of adenosine exposure, to 109.5 ± 2 % of control (*P* < 0.05, *n* = 8) (Fig. 4). The effect on the ON-fEPSP peak amplitude was not sustained but decayed prior to washout. A transient reduction in the ON-fEPSP was also observed before recovering fully, to 99.8 ± 2 % of control approximately 10 min later (*P* > 0.05), which is illustrated by the representative traces (Fig. 4c). Vehicle application had no significant effect on the ON-fEPSP (Fig. 4). These results suggest that the differential effects of BzATP and adenosine on the ON-fEPSP are due to the activation of different purinergic receptors.

### P2X_7_R activation modulates rod photoreceptor-ON bipolar cell synaptic transmission

**Fig. 5:**
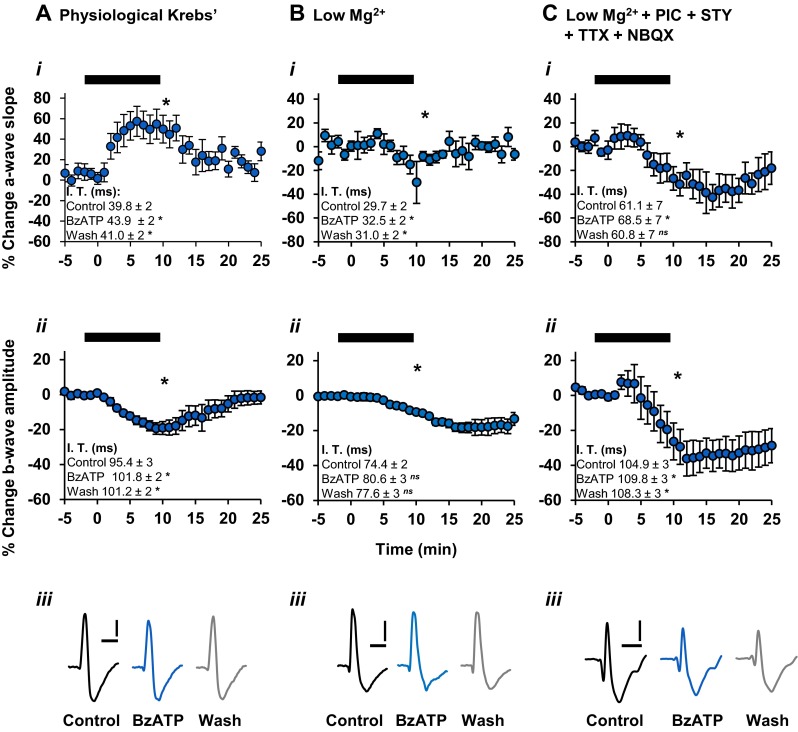
The effect of BzATP on the dark-adapted ERG when recorded under different conditions. Time course plots show the effect of BzATP (300 μM; 10 min) on the a-wave slope and b-wave amplitude (*bars*). Values are mean ± SEM percentage change and *insets* show mean ± SEM implicit time values. For all, *ns* not significant; *P < 0.05, compared to pre-treatment control. **a** In physiological Krebs medium (*n* = 13), BzATP induced a sustained potentiation of the a-wave slope (*i*), which reached maximal effect within approximately 5 min of application. BzATP concomitantly reduced the b-wave amplitude (*ii*) over the course of application, an effect which was recoverable following washout. **b** In the absence of extracellular magnesium ions (*n* = 6), BzATP reduced the a-wave slope (*i*) and also caused a reduction in the b-wave amplitude (*ii*) over the course of application, an effect which was not fully recoverable following washout. **c** In the absence of extracellular magnesium ions, and in the presence of NBQX, picrotoxin, strychnine and TTX (*n* = 7), BzATP reduced the a-wave slope (*i*). The b-wave amplitude was markedly suppressed (*ii*) and was not fully recoverable following washout. Note the relatively similar effect of BzATP on the b-wave, compared to that shown in **b**(*ii*). Representative traces (**a**(*iii*), **b**(*iii*), **c**(*iii*)) illustrate the effect of BzATP on the ERG under the respective conditions. *Scale bars* = 100 ms, 20 μV

The effect of P2X_7_R activation on synaptic transmission at rod photoreceptor-ON bipolar cell synapses was investigated under three different conditions. Dark-adapted retinal whole mounts were used to record visually evoked ERG responses. In physiological Krebs medium, a 10-min application of BzATP induced a marked potentiation of the a-wave slope to 149.0 ± 14 % of control (*P* < 0.05, *n* = 13) (Fig. 5a(i)). The BzATP-mediated changes in the a-wave were relatively rapid in onset, reaching a sustained maximum effect within approximately 5–6 min after the start of drug application. Following washout of BzATP, the a-wave slope recovered to 108.2 ± 3 % of control (*P* > 0.05). BzATP also significantly increased the implicit time of the a-wave maximum amplitude compared to pre-treatment control (*P* < 0.05) (Fig. 5a(i)).

A concomitant reduction of the ERG b-wave amplitude to 80.6 ± 4 % of control (*P* < 0.05, *n* = 13) was also measured in the presence of BzATP (Fig. 5a(ii)). The changes in the b-wave within 1–2 min of application appeared to reach maximum reduction in amplitude immediately prior to washout. Considering the direct output of the photoreceptors onto the ON bipolar cells along the vertical pathway, it would be expected that a potentiation in the a-wave slope would lead to a subsequent increase in the b-wave amplitude. However, this does not seem to be the case here, and is consistent with the in vivo findings of the effect of BzATP on the rat ERG [13]. The b-wave amplitude recovered to baseline within approximately 10 min following washout of BzATP (97.3 ± 3 % of control, *P* > 0.05). BzATP application also induced a potentiation in the implicit time of the b-wave (Fig. 5a(ii)). The differing effect of BzATP on the kinetics of the a-wave and b-wave, as has been previously demonstrated in vivo [13], suggests that there are independent photoreceptor and post-photoreceptor sites of action stimulated by the P2X_7_R agonist.

The effect of BzATP-mediated activation of P2X_7_Rs is considerably enhanced in the absence of extracellular divalent cations, particularly magnesium ions (Mg^2+^) [16, 40]. To further characterise the BzATP-mediated effect on the ERG response components, the P2X_7_R agonist was applied in normal physiological medium that did not contain magnesium ions. Under these conditions, BzATP induced a reduction in the a-wave slope to 90.7 ± 3 % of control (*P* < 0.05, *n* = 6), which was typically recoverable 5 min post-wash to 97.2 ± 3 % of control (*P* > 0.05) (Fig. 5b(i)). Furthermore, BzATP increased the implicit time of the a-wave maximum amplitude (Fig. 5b(i)). The BzATP-mediated reduction in the a-wave slope was contrary to that seen in the presence of extracellular magnesium, suggesting that the effect of P2X_7_R activation is reversed under these conditions.

In the absence of extracellular magnesium, BzATP significantly reduced the b-wave amplitude to 89.5 ± 1 % of control (*P* < 0.05, *n* = 6) (Fig. 5b(ii)). The temporal profile of the b-wave amplitude was comparable to that of the a-wave slope during the early phase of BzATP application, where an exponential reduction in both components was apparent. However, the differing recovery periods of the a- and b-waves strongly support the notion that BzATP elicited independent effects on photoreceptor and post-photoreceptor components. Additionally, BzATP did not elicit a significant effect on the implicit time of the b-wave maximum amplitude (Fig. 5b(ii)). The suppressive effect of BzATP on the b-wave amplitude in the presence and absence of extracellular magnesium was similar in magnitude. The differences in the changes to the b-wave amplitude during the post-wash phase under both conditions imply that magnesium was required for full recovery following BzATP application.

In separate experiments, the effects of BzATP on the ERG a-wave and b-wave components were investigated in the presence of the antagonists NBQX, picrotoxin, strychnine and tetrodotoxin and the absence of magnesium ions (PMSTN medium). As shown in Fig. 5c(i), there was a significant reduction in the a-wave slope to 70.1 ± 8 % of pre-treatment control after 5 min (*P* < 0.05, *n* = 7), with partial recovery following washout (76.3 ± 9 % of control, *P* < 0.05). The a-wave was also significantly delayed in the presence of BzATP, recovering on washout (Fig. 5c(i)). Furthermore, there was a significant difference in the BzATP-mediated effect on the a-wave slope compared to that observed in the low magnesium solution (*P* < 0.05).

The effect of BzATP on the b-wave amplitude, in the PMSTN bathing medium, followed a similar temporal profile to that of the a-wave slope, with a significant reduction to 66.6 ± 10 % of control (*P* < 0.05, *n* = 7) (Fig. 5c(ii)) with little recovery following washout of BzATP. The b-wave implicit time was also significantly increased with BzATP (Fig. 5c(ii)). Under these conditions, BzATP induced effects on the b-wave amplitude (*P* > 0.05) which were comparable to those seen in the absence of magnesium alone.

### BzATP-induced modulation of the ERG a-wave and b-wave was reduced by the selective P2X_7_R antagonist A438079

**Fig. 6:**
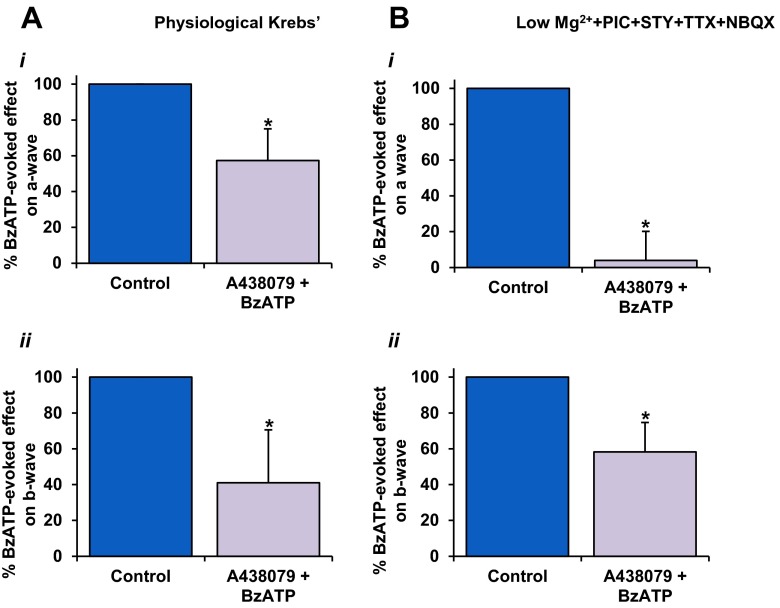
Actions of the selective P2X_7_R antagonist A438079 on the BzATP-mediated effects on the ERG components. Values are mean ± SEM percentage of control (max. BzATP effect, i.e. 100 %). **a** In physiological Krebs medium (*n* = 6), the BzATP-mediated effects on the a-wave slope (*i*) and b-wave amplitude (*ii*) were markedly reduced in the presence of A438079 (10 μM) compared to control. **b** In the absence of extracellular magnesium ions, and in the presence of NBQX, picrotoxin, strychnine and TTX (*n* = 7), A438079 almost completely suppressed the BzATP-mediated potentiation of the ERG a-wave compared to control. **b** A438079-induced attenuation of the BzATP-mediated reduction in the b-wave was also observed. *P < 0.05, compared to pre-treatment control

**Fig. 7:**
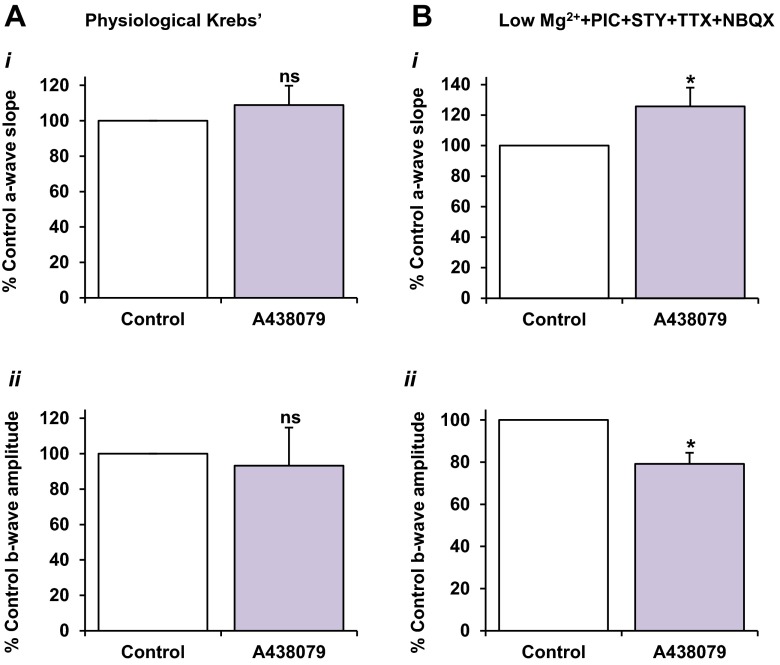
The direct effect of the selective P2X_7_R antagonist A438079 on ERG components under different physiological conditions. Values are mean ± SEM percentage of pre-treatment control; *n* = 10. A438079 (10 μM) was superfused for 20–30 min until the response was stable. **a** In physiological Krebs medium, application of A438079 alone had no overall effect on the a-wave (*i*) or b-wave (*ii*). **b** In separate experiments using the pharmacologically modified Krebs medium, A438079 induced a marked potentiation in the a-wave (*i*) compared to control (*P* < 0.05). The b-wave (*ii*) was significantly reduced in the presence of A438079 (*P* < 0.05)

In order to further delineate P2X_7_R involvement, the effect of BzATP on the ERG components was assessed in the presence of the selective, competitive P2X_7_R antagonist, A438079 (10 μM). The effect of A438079 on the BzATP-induced changes in the a- and b-waves was directly compared to the preceding application of BzATP in the same experiment. When tested in physiological Krebs medium, A438079 significantly reduced the potentiating effect of BzATP on the a-wave slope to 57.3 ± 18 % of the effect of BzATP when applied alone (*P* < 0.05, *n* = 6) (Fig. 6a(i)). Similarly, the BzATP-mediated suppression of the b-wave was markedly reduced in the presence of the antagonist to 41.0 ± 30 % of control (*P* < 0.05, *n* = 6) (Fig. 6a(ii)). Next, the effect of the P2X_7_R antagonist on the action of BzATP was tested in PMSTN medium. As shown in Fig. 7b(i), A438079 abolished the BzATP-mediated reduction of the a-wave slope to 4 ± 16 % of the effect of BzATP when applied alone (*P* < 0.05, *n* = 7). Furthermore, A438079 reduced the effect of BzATP on the b-wave amplitude to 58.3 ± 16 % of control (maximum BzATP effect; *P* < 0.05) (Fig. 6b(ii)). Thus, the A438079-mediated attenuation in the effect of BzATP on these components of the ERG confirms the involvement of P2X_7_R activation.

### The effect of direct application of the P2X_7_ receptor antagonist A438079 on the dark-adapted electroretinogram a-wave and b-wave components

To explore whether the release of endogenous ATP acting on P2X_7_Rs during visual stimulation affects outer retinal processing, the effect of A438079 on the ERG when applied alone was investigated. In physiological Krebs medium, A438079 (10 μM) alone did not induce an effect on the a-wave (108.9 ± 10 % of control, *P* > 0.05, *n* = 9) (Fig. 7a(i)). Similarly, the b-wave also remained unaffected by the application of the antagonist (93.3 ± 22 %, *P* > 0.05, *n* = 9) (Fig. 7a(ii)). Conversely, in the pharmacologically modified perfusion medium, the a-wave slope was significantly potentiated in the presence of A438079 (10 μM) alone, to 125.7 ± 12 % of pre-treatment control (*P* < 0.05, *n* = 10). A438079 also markedly reduced the b-wave amplitude to 79.1 ± 15 % of control (*P* < 0.05, *n* = 10). The implicit times for both a- and b-waves were increased in the presence of the antagonist. These results suggest that P2X_7_R activation may modulate retinal function under baseline conditions but that this depends on the particular ionic conditions of the microenvironment.

### ERG oscillatory potentials are suppressed by P2X_7_R activation

The effect of P2X_7_R activation on the ERG oscillatory potentials was also explored, using physiological Krebs medium. The high-frequency oscillatory potentials were digitally extracted from the raw ERG waveform (see Fig. 2). Changes in the peak power output of the oscillatory potentials were assessed with their frequency spectra before, during and after application of the pharmacological compound. As seen with the a-wave and b-wave components recorded in physiological Krebs medium, A438079 alone had no significant effect on the peak power of the oscillatory potentials (124.6 ± 25 % of control, *P* > 0.05, *n* = 6).

**Fig. 8:**
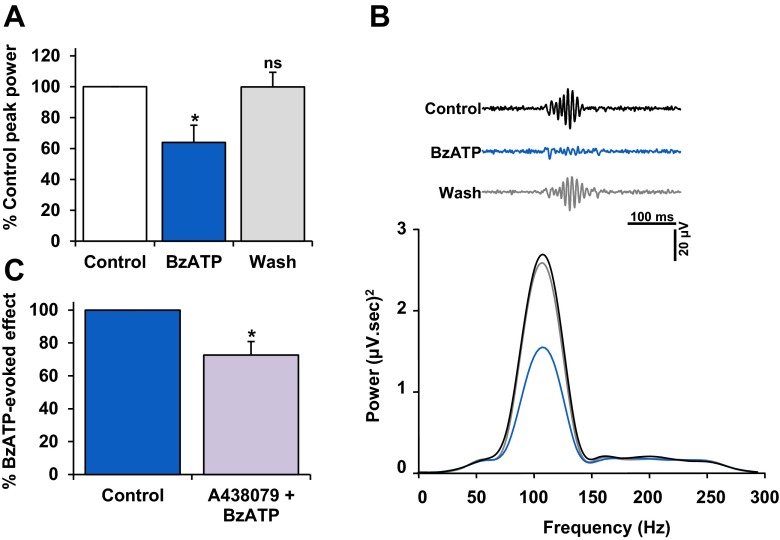
The effect of BzATP on ERG oscillatory potentials. Experiments were done in physiological Krebs medium. **a** Mean ± SEM percentage of control peak power; *n* = 7. BzATP (300 μM; 10 min) caused a reversible and significant reduction in the peak power output of the oscillatory potentials compared to control (*P* < 0.05), indicating modulation of inner retinal function. **b**(*i*) Representative oscillatory potentials, before, during and post-BzATP application. *Black*, control; *blue*, BzATP; *grey*, wash. Under control conditions, the oscillatory potentials exhibited a peak frequency ∼107 Hz, typical of dark-adapted ERG responses. In the presence of BzATP, no overall shift in frequency was detected. **b**(*ii*) Power Spectra (120 s) illustrate the BzATP-mediated suppression of the oscillatory potentials and recovery on washout. *Black* control; *blue*, BzATP; *grey*, wash. **c** A438079 (10 μM) significantly reduced the BzATP-evoked suppression of the oscillatory potentials compared to control. *P < 0.05, compared to pre-treatment control

BzATP significantly reduced the peak power to 58.8 ± 8 % of control (*P* < 0.05, *n* = 13) (Fig. 8a, b). Following washout of BzATP, the peak power of the oscillatory potentials recovered to 97.9 ± 10 % of control (*P* > 0.05). Next, the effect of A438079 was tested on the BzATP-induced suppression of the oscillatory potentials. A438079 significantly reduced the effect of BzATP on the oscillatory potential peak power to 68.2 ± 8 % of control (*P* < 0.05, *n* = 6) (Fig. 8c). These results suggest that BzATP considerably suppressed the activity of inner retinal networks, and further imply that P2X_7_R modulation in the inner retina is at least partially independent of that in the outer retina.

## Discussion

### P2X_7_Rs modulate the ON pathway in the outer and inner retina

The present study has demonstrated that neuronal function in both the outer and inner retina of the mouse under dark-adapted conditions is considerably altered by P2X_7_R activation. An overview of the key findings are summarised in Table 1. Furthermore, P2X_7_R modulation of visual responses appears to occur through independent mechanisms, at different levels of the rod pathway. These findings are not unexpected as P2X_7_Rs are expressed on photoreceptors; horizontal, amacrine and retinal ganglion cells; and on non-neuronal cells including microglia, retinal pigment epithelial cells and vascular endothelial cells within the retina. As of yet, P2X_7_Rs have not been localised to bipolar cells.

**Table 1:** Summary of the effect of BzATP on the RGC ON-fEPSP and ERG components in the absence and presence of P2X_7_R antagonists under the physiological conditions tested. All values are expressed as mean ± SEM. See text for *n* and *P* values. The effect of BzATP alone is compared to pre-treatment control. The effect of BzATP in the presence of an antagonist is compared to the maximum BzATP-mediated effect

	Physiological Krebs	Modified Krebs (PMSTN)	Low Mg^2+^
RGC ON-fEPSP	–	BzATP: 78.1 ± 3 %	–
		BzATP + antagonist (% of max. BzATP effect)	
		A438079: 68.7 ± 2 %	
		A804598: 55.1 ± 11 %	
		AF27139: 75.2 ± 7 %	
ERG a-wave	BzATP: 149.0 ± 14 %	BzATP: 70.1 ± 8 %	BzATP: 90.7 ± 3 %
	BzATP + A438079: 57.3 ± 18 %	BzATP + A438079: 4 ± 16 %	–
ERG b-wave	BzATP: 80.6 ± 4 %	BzATP: 66.6 ± 10 %	BzATP: 89.5 ± 1 %
	BzATP + A438079: 41.0 ± 30 %	BzATP + A438079: 58.3 ± 16 %	–
ERG OPs	BzATP: 58.8 ± 8 %	–	–
	BzATP + A438079: 68.2 ± 8 %		

*OPs* oscillatory potentials

### Modulation of outer retinal function

P2X_7_R-mediated alterations to synaptic transmission from rod photoreceptors to rod-ON bipolar cells were assessed using the dark-adapted ERG. Under physiological conditions, P2X_7_R activation by its agonist, BzATP, potentiated the a-wave, with a concomitant reduction in the b-wave. This finding is consistent with previous work in the rat in vivo [13]. BzATP also delayed the time to peak of the a-wave, which could indicate a reduction in photosensitivity of the rods [41]. However, in the study by Puthussery et al. [13], computational extraction of the sensitivity parameter of the a-wave leading edge indicated no change in photoreceptor sensitivity as a result of P2X_7_R activation. Thus, the change in implicit time of the a-wave in this investigation could instead be attributed to a possible contamination of the BzATP-induced effect on the b-wave.

The opposing effects of BzATP on the photoreceptor and bipolar cell response components could be explained by a mechanism associated with a change in the dark current of the photoreceptors. Depolarisation of the photoreceptor terminal induced by P2X_7_R activation would subsequently potentiate the a-wave during photostimulation, due to a greater change in the photoreceptor dark current, as has been suggested previously [13]. Concurrently, facilitation of the photoreceptor dark current would increase the rate of glutamate release in the dark, thus eliciting a smaller light-induced depolarisation of the ON BCs. Stable calcium influx through L-type channels is imperative for the sustained release of glutamate from rod terminals in darkness [42]. A P2X_7_R-dependent rise in intracellular calcium has previously shown to directly stimulate vesicular glutamate release from hippocampal and cortical presynaptic terminals [20–22]. The present findings suggest that P2X_7_R activation may modulate ERG responses through a similar calcium-dependent mechanism, although direct P2X_7_R modulation of ribbon synapses has not yet been shown.

P2X_7_R activity is altered and most likely enhanced in the absence of extracellular magnesium [16, 40]. In the present study, removal of magnesium ions from the perfusate reversed the effect of P2X_7_R activation on the a-wave, instead inducing a marked reduction in the photoreceptor response. This supports the concept of a bi-directional function of P2X receptors in modulating synaptic activity dependent upon the physiological context, as has been consistently evident in central synapses, particularly in the hippocampus [43]. Without extracellular magnesium, the b-wave was suppressed by BzATP, to a similar magnitude, when compared to the effect in the presence of the divalent cation. Contrary to the observed effect in physiological perfusate, the BzATP-mediated suppression of the b-wave was not reversible in the absence of magnesium. The differential temporal profiles of the effect of BzATP on the a- and b-waves highlight independent sites of modulation by P2X_7_Rs in both the inner and outer retina.

In the present study, the suppressive effect of P2X_7_R activation on the photoreceptor response was enhanced in the presence of blockers of GABA_A/C_, glycine and AMPA/KA receptors and of voltage-gated sodium channels. It has previously been suggested that P2X_7_R-associated modulation of the mouse ERG may be due to alterations in horizontal and amacrine cell feedback [7]. However, the underlying mechanism of modulation of the photoreceptor response may not be associated with the release of inhibitory inputs to the rod terminals under dark-adapted conditions [44]. Indeed, the study by Vessey and Fletcher [7] found that the ERG a-wave was unaltered, whereas the b-wave was markedly potentiated in a P2X_7_R-knockout mouse model. In the present investigation, there was no overall difference in the BzATP-induced reduction in the b-wave, between responses recorded in the absence of magnesium alone, and with the addition of pharmacological blockers of inhibitory neurotransmission. This indicates that P2X_7_R-mediated modulation of the ON BCs is not due to an effect on inhibitory feedback at the ON BC terminal in the inner retina. Since BCs do not themselves express P2X_7_Rs, these results could reflect a possible modulation of both ERG components by non-neuronal cells during P2X_7_R stimulation.

The involvement of P2X_7_R-mediated modulation of the a-wave was confirmed with the selective antagonist A438079, which markedly suppressed and abolished the effect of P2X_7_R activation on the photoreceptor response, when tested in physiological Krebs and the pharmacologically modified medium, respectively. Similarly, A438079 partially blocked P2X_7_R modulation of the ON bipolar cell response under both conditions tested. The presence of a residual effect of BzATP even with the addition of a selective P2X_7_R antagonist supports the notion that a consistent or secondary stress-related release of large amounts of endogenous ATP, possibly by non-neuronal cells, could act to influence retinal synaptic signalling.

To explore whether P2X_7_Rs have a physiological role in outer retinal function upon visual stimulation, we investigated the effects of A438079 alone on the ERG components. When recorded in the physiological Krebs medium, blockade of P2X_7_Rs had no overall effect on the a-wave or b-wave. This contradicts previous findings whereby the b-wave was significantly potentiated in a P2X_7_R knockout model [7]. However, both ERG components were markedly suppressed when the antagonist was applied in the modified perfusion medium. A sensitised or stressed cellular microenvironment can initiate acute changes to glutamate and ATP homeostasis and signalling. Such deviations from the physiological norm may increase the likelihood of ATP release in the outer retina, at a sufficient concentration to activate P2X_7_Rs and subsequently affect the processing of the visual signal.

These results strongly suggest a direct role for P2X_7_Rs in photoreceptors in modulating outer retinal processing and are supported by studies that have shown P2X_7_R immunoreactivity at ribbon synapses within the rod spherule, in the rodent retina [7, 12, 13]. However, P2X_7_R modulation of photoreceptor function through alternative mechanisms cannot be ruled out. The P2X_7_R expression profile in the healthy rabbit [9] and human retina [36, 45] has shown no evidence of immunoreactivity in photoreceptors and may instead support the concept that P2X_7_Rs may modulate photoreceptor function through its expression in retinal pigment epithelial cells in the absence of pathology. Indeed, RPE cells are sources of ATP, which can be released in response to hyperosmotic stress [46], through various mechanisms including ATP-triggered secondary ATP release, CFTR-dependent ATP efflux as well as vesicular ATP secretion, as demonstrated in human retinal cells [47]. This autostimulatory ATP release may be driven through P2X_7_R-associated panx-1 hemichannels, as shown in the bovine retina [48]. Furthermore, RPE cells have demonstrated the presence of ectonucleotidases [46], which may rapidly degrade ATP into its constituents, in order to activate other P2 and adenosine receptors in cells of the outer retina, and therefore influence synaptic signalling through alternative mechanisms.

### Modulation of inner retinal function

The present study has demonstrated that P2X_7_R activation distinctly influences the activity of inner retinal networks, as shown by the changes observed in the ERG oscillatory potentials. A clear effect of P2X_7_R activation on the ON pathway was apparent, as BzATP suppressed the ON-fEPSP in a concentration-related manner. The involvement of P2X_7_Rs in modulating the ON-fEPSP was supported by the partially attenuated effect of BzATP in the presence of the selective P2X_7_R antagonists A438079, A804598 or AF27139. With each antagonist, a residual effect of BzATP on the ON-fEPSP remained, which could be explained by a lack of potency of these compounds for blocking P2X_7_R-mediated effects in the inner retinal layers.

As with the outer retina, the lack of potency of these compounds in suppressing the BzATP-mediated reduction in ON-centre RGC responses could be due to the stimulated release of endogenous ATP within the inner retinal layers. ATP is present in high concentrations within all metabolically active cells and so all can function as sources of ATP, which may then act to compete with the antagonist at the receptor. Alternatively, P2X_7_Rs are known to drive ATP release which can then act cell-autonomously. Indeed, P2X_7_R-mediated, non-vesicular release of ATP has been demonstrated in retinal ganglion cells [9]. P2X_7_Rs are expressed on amacrine, ganglion and microglial cells in the inner mouse retina and are all therefore potential sources of ATP-mediated ATP release.

In the absence of pathology or inflammation, the expression of functional P2X_7_Rs has only been demonstrated in Müller cells of the human retina but not in those of other mammalian species [49]. However, it has been shown in the rat retina that light-evoked ATP release from ganglion cells increases Müller cell Ca^2+^ transients through activation of glial P2YRs. This in turn mediates the release of glial ATP, which is rapidly hydrolysed into adenosine, in turn hyperpolarising ganglion cells through the activation of A1 receptors [50]. It has been proposed that the BzATP-mediated suppression of excitatory responses in the rodent hippocampus and brainstem was attributed to its breakdown product Bz-adenosine and subsequent activation of adenosine receptors [39, 51]. Adenosine facilitated the ON-fEPSP for a short period before the effect diminished, leading to a temporary suppression of the ON-fEPSP before recovery on washout. This may be due to a number of factors, which include receptor desensitisation or clearance of adenosine from the extracellular environment via nucleoside transporters expressed in Müller cells. In the present study, the possible contribution of A1 receptors to the BzATP-mediated reduction of the ON-fEPSP was ruled out. Adenosine produced a dose-related potentiation in the ON-fEPSP, an effect which followed a differential temporal profile to that seen for the effect of BzATP. This suggests that adenosine facilitates possibly through A3 receptors, whereas P2X_7_Rs appear to reduce neuronal activity within the ON pathway.

Although BzATP is a potent agonist of P2X_7_Rs, it is not selective for the receptor and can bind to P2X_1_, P2X_2_ and P2X_3_ receptors with similar potency [17], and therefore, the possibility remains that these receptor subtypes may also be activated in the presence of the agonist. P2X_1_R immunoreactivity has been localised to the inner retinal layers of the cat and monkey retina [52], although evidence of its expression and function in the mouse is uncertain. In the rodent, P2X_3_Rs have been detected in the inner plexiform layer [53]. On activation, both P2X_1_Rs and P2X_3_Rs rapidly desensitise [1], and thus their kinetic profiles do not correlate with the BzATP-mediated effects observed in the present study. Selective expression of P2X_2_Rs in starburst amacrine cells within the OFF vertical pathway has been demonstrated in the mouse retina [54, 55]. Although P2X_2_Rs display slowly desensitising properties similar to P2X_7_Rs, it is uncertain whether they would directly affect neuronal responses within the ON pathway.

Metabotropic P2Y receptors may also modulate visual responses in the retina as they have been suggested to play a role in glial-neuronal cross-talk [48]. Indeed both functional and immunohistochemical expression of P2Y_1_Rs on Müller cells [11, 48, 56, 57] and P2Y_4_R on rod bipolar and amacrine cells [58] have been demonstrated in the retina. Considering that ADP and UTP are markedly more potent agonists at these receptors, respectively [59], their direct contribution to the BzATP-mediated effect on visual responses in the present study is unlikely. However, it is important to note that the remarkable diversity of purinergic receptors in such an intimate retinal microenvironment opens the possibility of their contribution to the overall glial-neuronal cross-talk in the processing of visual information, through the secondary release of endogenous purinergic ligands.

The present findings are in accord with previous findings [8, 10] on P2X_7_R function in the retina and extend these findings to show that activation of these receptors can affect function at different sites in the retinal ON pathway. Thus, activation of these receptors under pathophysiological conditions by high concentrations of ATP would likely affect function at all stages of visual transmission in the retina.
